# The Association between Resilience and Psychological Distress during the COVID-19 Pandemic: A Systematic Review and Meta-Analysis

**DOI:** 10.3390/ijerph192214854

**Published:** 2022-11-11

**Authors:** Thanakrit Jeamjitvibool, Cherdsak Duangchan, Andria Mousa, Wiriya Mahikul

**Affiliations:** 1 Faculty of Nursing, HRH Princess Chulabhorn College of Medical Science, Chulabhorn Royal Academy, Bangkok 10210, Thailand; 2 College of Nursing, University of Illinois at Chicago, Chicago, IL 60612, USA; 3 Department of Infection Biology, London School of Hygiene and Tropical Medicine, London WC1E 7HT, UK; 4 Princess Srisavangavadhana College of Medicine, Chulabhorn Royal Academy, Bangkok 10210, Thailand

**Keywords:** resilience, psychological distress, COVID-19, general population, healthcare workers, patients

## Abstract

This study examined the association between resilience and psychological distress in healthcare workers, the general population, and patients during the COVID-19 pandemic. We searched the PubMed, Web of Science, PsycInfo, Science Direct, and Nursing and Allied Health databases. Included articles examined healthcare workers (e.g., physicians and nurses), the general population, and patients during the COVID-19 pandemic. Studies of exposure to other infectious diseases related to epidemics or pandemics (e.g., SARS and MERS) were excluded. This study was performed following the Cooper matrix review method and PRISMA guidelines, followed by a meta-analysis of study results using R version 4.1.2. A random effect model was used for the pooled analysis. This study was registered with PROSPERO (registration No. CRD42021261429). Based on the meta-analysis, we found a moderate negative relationship between overall resilience and psychological distress (r = −0.42, 95% confidence interval [CI]: −0.45 to −0.38, *p* < 0.001). For the subgroup analysis, a moderately significant negative relationship between overall resilience and psychological distress was found among healthcare workers (r = −0.39, 95% CI: −0.44 to −0.33, *p* < 0.001), which was weaker than in the general population (r = −0.45, 95% CI: −0.50 to −0.39, *p* < 0.001) and in patients (r = −0.43; 95% CI: −0.52 to −0.33; *p* < 0.001). This association was robust, although the heterogeneity among individual effect sizes was substantial (I^2^ = 94%, 99%, and 74%, respectively). This study revealed a moderate negative relationship between resilience and psychological distress in healthcare workers, the general population, and patients. For all these populations, interventions and resources are needed to improve individuals’ resilience and ability to cope with psychological distress during the COVID-19 pandemic and in future disease outbreaks.

## 1. Introduction

The rapid worldwide spread of coronavirus disease 2019 (COVID-19) has dramatically impacted various aspects of global public health. This disease, which affects the respiratory system and is easily transmitted from one person to another, can be fatal in vulnerable populations. On 20 June 2021, the World Health Organization (WHO) reported that the first wave of COVID-19 infections had reached approximately 177 million people and that 3.8 million people had died from the disease both in the general population and among healthcare providers [1].

In addition to its physiological impacts, the COVID-19 pandemic has adversely affected mental health, leading to psychological distress in populations across the globe [2,3]. Psychological distress is defined as the reaction of an individual to external and internal stresses and as a mixture of psychological symptoms encompassing stress, depression, and anxiety [4,5]. During COVID-19, the prevalence of psychological distress was higher than before the pandemic [6,7,8,9,10,11,12,13,14]. According to a recent systematic review and meta-analysis, during the COVID-19 pandemic, the pooled incidence of depression, anxiety, insomnia, post-traumatic stress disorder (PTSD), and psychological distress was 16.0%, 15.5%, 23.9%, 21.9%, and 13.3%, respectively [15]. The factors associated with these mental health disorders included the loss of family members and friends to COVID-19 [16] as well as preventive measures implemented to reduce the spread of the disease, such as lockdowns of areas or whole countries [17], social distancing from family and friends [18], and loss of employment due to economic impacts [19].

In particular, frontline healthcare providers working during the COVID-19 pandemic experienced many psychological problems. For example, they faced anxiety due to COVID-19 infections and mental health problems in family members [20]. Additionally, insufficient knowledge and guidelines, lack of medical equipment and supplies, and staff shortages are likely to have resulted in depression as well as anxiety among providers [15,21,22]. One study conducted during the first wave of COVID-19 found that younger age, lower levels of education, and lower economic status were all significantly associated with mental health problems [8]. Other studies during the pandemic reported that more than 20% of nurses [7], healthcare providers overall [21], and the general population [15] were affected by psychological distress, which was significantly associated with less social support and lower resilience.

During the stressful situations of the COVID-19 pandemic, previous studies reported a negative relationship between psychological distress and psychological resilience. Resilience refers to the ability to cope with adversity and to adapt to major life events [23,24]. This ability varies widely from person to person and depends on environmental as well as personal factors [23,24]. Wagnild and Young [25] found that resilience was positively correlated with adaptational outcomes such as physical health and life satisfaction and negatively correlated with psychological distress. Moreover, Verdolini et al. [2] found that psychological resilience had a significant negative relationship with psychological distress during the pandemic. Furthermore, survey studies showed that resilience had a negative association with psychological distress among physicians [26] and with depressive symptoms in the general population [27]. Factors contributing to resilience were found to include greater age and higher educational level [28].

Overall, the prevalence of mental health problems and the presence of resilience have both been assessed in the general population and among healthcare providers during the COVID-19 pandemic [6,7,8]. Previous systematic reviews have examined the relationship between psychological distress and psychological resilience in chronic illness patients [29] and the general population [30] before the COVID-19 pandemic. To date, however, no systematic review and meta-analysis have been conducted to explore the relationship between resilience and psychological distress, including depression, stress, anxiety, and PTSD, in healthcare providers, the general population, and patients during the first wave of the pandemic, all of which can affect other aspects of mental health [31,32]. Several studies published in 2022 documented the effects of the pandemic on mental health [33,34,35], and others examined resilience [36,37]. However, no systematic review and meta-analysis assessing the correlation between resilience and psychological distress have been published. Therefore, this study aimed to employ correlational coefficients to explore the relationship between resilience and psychological distress in the three populations during the first wave of the pandemic and evaluate whether this relationship differed among healthcare workers, the general population, and patients. The findings are expected to promote an understanding of psychological distress and how resilience can be promoted to help manage such distress during both the present COVID-19 pandemic and future disease outbreaks.

## 2. Materials and Methods

### 2.1. Search Strategy

This study was registered in PROSPERO (registration No. CRD42021261429) to avoid duplication of effort and minimize the chance of reporting bias. The systematic review was conducted following Preferred Reporting Items for Systematic Reviews and Meta-Analyses (PRISMA) guidelines [38]. PubMed, Web of Science, PsycInfo, Science Direct, and the Nursing and Allied Health Database were used to search for studies for this review on 2 June 2021; the search strategy, including keywords and index terms, was adapted as necessary for each database. In addition, the reference list of each included source of evidence was screened to identify potential additional studies. Because COVID-19 first emerged in Wuhan, China, in December 2019, and because this study was limited to the first wave of the pandemic, only studies published from 1 December 2019 to 1 June 2021 were included in the review. Population-intervention-comparison-outcome (PICO) keywords applied during the database search are shown in Supplementary Table S1.

### 2.2. Selection Criteria

The inclusion criteria for the study were as follows: (1) a full-text journal article published in English; (2) original quantitative research focusing on the relationship between resilience and psychological distress, including depression, stress, anxiety, and PTSD, using correlational coefficients; and (3) use of self-reported measurement of resilience with the Connor–Davidson Resilience Scale (CD-RISC), which is the scale most widely used to assess psychological resilience. This instrument focuses on resources that can help individuals to recover from and adapt to disruptions or stressful events such as the COVID-19 crisis. We did not include studies using the Brief Resilience Scale (BRS), which directly measures one’s ability to bounce back or be resilient but does not consider external resources [39]. In addition, although other resilience instruments have been applied in resilience studies, the various theoretical constructs and frameworks of resilience underpinning these instruments were not suitable for conducting our meta-analysis due to heterogeneity issues. Furthermore, we excluded sources if they (1) reported studies of other infectious diseases related to epidemics or pandemics (e.g., SARS or MERS); (2) were review or interventional studies; or (3) were gray literature, books, abstracts, or study protocols.

### 2.3. Study Selection

**Figure 1:**
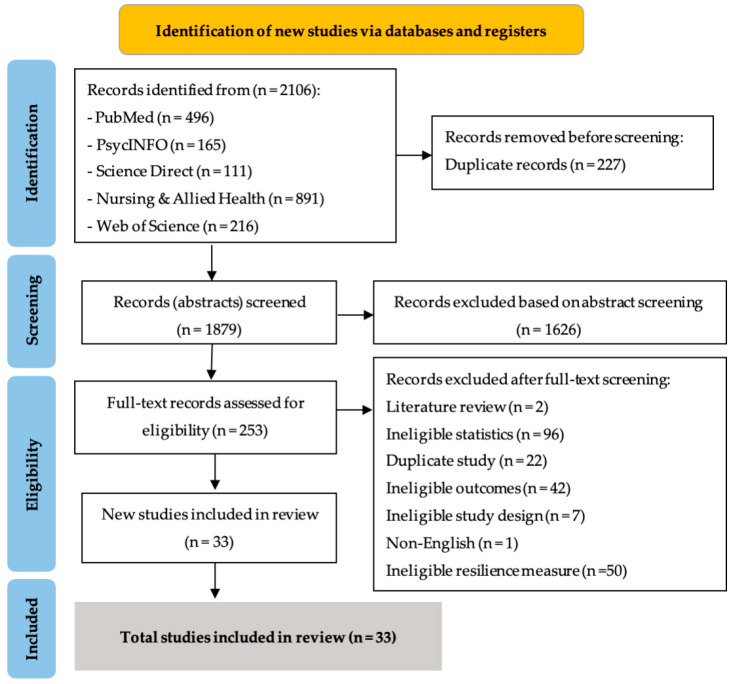
PRISMA flow diagram for systematic review.

Following the literature search, all identified studies (N = 2106) found in PubMed (n = 496), Web of Science (n = 216), PsycINFO (n = 165), Science Direct (n = 111), and the Nursing and Allied Health Database (n = 891) were exported into EndNote X9 reference management software [40]. After duplicates were removed (n = 227), the 1879 remaining studies were exported into the Joanna Briggs Institute System for the Unified Management, Assessment, and Review of Information (JBI SUMARI) [41]. The researchers (TJ and WM) then independently screened their titles and abstracts in accordance with JBI SUMARI procedures based on selection criteria such as the publication language, participants, study design, and use of the CD-RISC self-report measure. After screening, 1626 ineligible records were excluded. The full texts of the 253 remaining articles were then retrieved for eligibility screening. During both title/abstract and full-text screening, discrepancies between two authors’ (TJ and WM) independent assessments were generally resolved through discussion. Finally, a total of 33 studies meeting the eligibility criteria were included in the review. The study selection process and reasons for excluding particular studies are shown in Figure 1.

### 2.4. Quality Appraisal

The Joanna Briggs Institute (JBI) critical appraisal checklist for analytical observational studies [42] was used to assess the methodological quality of the included studies. This tool is specifically designed for the assessment of cross-sectional studies. The checklist includes eight items for appraisal of the following: clarity of inclusion criteria, an adequate description of the study subject and setting, validity and reliability of measurement, whether measurements of conditions were objective and standardized, identification of confounding factors, strategies to account for confounders, validity of outcome measurement, and appropriate use of statistics. The JBI checklist for cohort studies consists of eleven items addressing the representativeness of included participants, the representativeness and validity of exposure measurement, whether and how confounding factors were adjusted for the validity of outcome measurement, whether participants were outcome-free at the start of the study, the adequacy and completeness of follow-up, the strategies used to address incomplete follow-up, and the appropriate use of statistics. In both checklists, the following four options are provided for each item: “Yes”, “No”, “Unclear” and “Not applicable”. The total checklist score ranges from 0 to 8 for cross-sectional studies and from 0 to 11 for cohort studies, with a higher score indicating higher quality; however, no cutoff point is provided to definitively identify the quality of studies [43].

### 2.5. Data Extraction

Data were independently extracted from the studies by TJ and WM. Cooper’s review matrix method was employed to assist in the analysis of the data [44]. We summarized the following data from the studies: author(s)/year, sample, country, the CD-RISC version (with reliability or internal consistency information expressed as Cronbach’s alpha), number of participants, and correlation coefficients. In addition, study data regarding measures of psychological distress (depression, stress, anxiety, and PTSD), their reliability range, and the number of associations identified (K) were summarized.

### 2.6. Meta-Analysis

R software version 4.1.2 was used for all analyses. The pooled correlation coefficient between resilience and psychological distress was calculated using the values of correlation coefficients obtained in each study and employing the “metacor” package [45]. Correlation coefficient values were generated along with 95% confidence intervals (CI). A random-effects model was used for the pooled analysis to account for unmeasured heterogeneity between studies. Correlations were classified as poor (correlation coefficient *r* < 0.20), average (*r* = 0.20–0.39), moderate (*r* = 0.40–0.59), strong (*r* = 0.60–0.79), and very strong (*r* ≥ 0.80) [46]. Publication bias was visually assessed using Begg’s funnel plots generated by the “metabias” package [47] and statistically assessed using Egger’s test in the “funnel.meta” package. The heterogeneity of *r* values between studies was tested by estimating a Cochran’s Q statistic and an inconsistency index (I^2^ statistic), with I^2^ > 50% indicating substantial heterogeneity. Where heterogeneity was substantial, a subgroup analysis, which is superior to meta-regression [48], was performed for all the studies to further investigate the heterogeneity issue. 

## 3. Results

### 3.1. Study Characteristics

The 33 selected studies were all published between 2019 and 2021. Of these studies, 31 had cross-sectional designs, and two were cohort studies. The studies were conducted in China (n = 18) [1,49,50,51,52,53,54,55,56,57,58,59,60,61,62,63,64,65], Spain (n = 3) [66,67,68], Israel (n = 2) [69,70], Iran (n = 1) [71], the Philippines (n = 1) [72], Japan (n = 1) [73], South Korea (n = 1) [74], the United States (n = 1) [75], South Africa (n = 1) [76], Portugal (n = 1) [77], Slovenia (n = 1) [78], Brazil (n = 1) [79], and Indonesia (n = 1) [80] (Table 1). The pooled sample size across the included studies was 34,366, with samples ranging from 60 to 7800 participants in individual studies. Most studies were conducted with healthcare personnel, including healthcare workers (n = 8) [51,53,56,57,59,61,73,80], nurses (n = 6) [50,52,58,60,71,74], resident physicians (n = 1) [67], first-line rescuers (n = 1) [49], and physiotherapists (n = 1) [77]. The remaining studies’ samples consisted of adults in the general population (n = 9) [1,66,68,69,70,72,75,78,79], college students (n = 4) [54,62,63,64], and patients (n = 3) [55,65,76].

### 3.2. Quality Appraisal

Two authors (TJ and WM) independently appraised the quality of the 33 included studies using two versions of the JBI critical appraisal checklist based on the study design. For the 31 cross-sectional studies, scores ranged from 4 to the maximum of 8 (median score: 8/8, interquartile range: 7/8–8/8; Supplementary Table S2). The two cohort studies scored 7 and 11 (Supplementary Table S3). For 10 studies, the two authors (TJ and WM) assigned inconsistent scores; consequently, another author (CD) reviewed the scores and assisted them in reaching a consensus. The results of the quality appraisal are detailed in Supplementary Tables S2 and S3.

### 3.3. Resilience

The included studies measured resilience, an assessment of stress coping ability employed to target treatment for anxiety, depression, and stress reactions, using various versions of CD-RISC [81]. Specifically, studies employed the 25-item (n = 16) [1,50,51,53,54,56,57,58,59,60,65,71,76,77,79,80], 10-item (n = 16) [49,52,55,61,63,64,66,67,68,69,70,72,73,74,75,78], and 27-item (n = 1) [62] versions of this measure. The reliability of the instruments (Cronbach’s α) ranged from 0.64 to 0.98 for the 25-item version and from 0.85 to 0.95 for the 10-item version; one study reported a Cronbach’s α of 0.86 for the 27-item version (Table 1).

### 3.4. Psychological Distress

The scales used to measure the four psychological distress variables, their range of reliability, and the number of associations with resilience (k) identified using each scale is shown in Supplementary Table S4. Most of the studies measured psychological distress using surveys containing scales for mental illness (k = 4), depression (k = 18), anxiety (k = 22), stress (k = 14), and PTSD (k = 2).

### 3.5. Relationship between Resilience and Psychological Distress

Based on this meta-analysis, a moderate negative relationship was detected between resilience and psychological distress (r = −0.42; 95% CI: −0.45 to −0.38; *p* < 0.001). Studies assessing this relationship showed high heterogeneity in their outcomes (I^2^ = 97.7%). Findings regarding associations between resilience and psychological distress are summarized in Table 1.

### 3.6. Subgroup Analysis

#### 3.6.1. Healthcare Workers 

**Figure 2:**
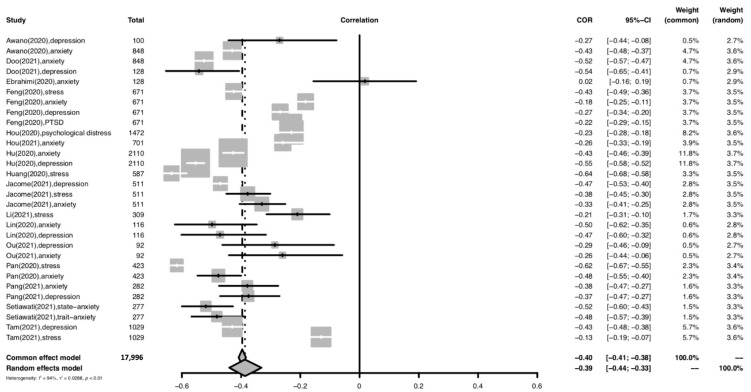
Forest plot of the relationship between resilience and psychological distress among healthcare workers. Abbreviation: COR, correlation coefficient; CI, confidence interval; I^2^, indicator of statistical heterogeneity; *p*, *p*-value; $\tau^{2}$, indicator of statistical heterogeneity. Horizontal lines indicate the 95% CI of each study; diamonds indicate the pooled estimate with a 95% CI.

The forest plot for meta-analysis of the relationship between resilience and psychological distress among healthcare workers is provided in Figure 2. For the healthcare worker subgroup, a moderately significant negative relationship was found between overall resilience and psychological distress (r = −0.39; 95% CI: −0.44 to −0.33; *p* < 0.001). The heterogeneity of the effect sizes was high (I^2^ = 94%).

#### 3.6.2. General Population 

**Figure 3:**
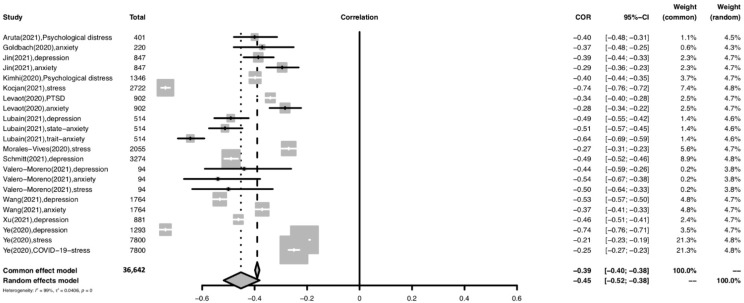
Forest plot of the relationship between resilience and psychological distress among the general population. Abbreviation: COR, correlation coefficient; CI, confidence interval; I^2^, indicator of statistical heterogeneity; *p*, *p*-value; $\tau^{2}$, indicator of statistical heterogeneity. Horizontal lines indicate the 95% CI of each study; diamonds indicate the pooled estimate with a 95% CI.

The forest plot for meta-analysis of the relationship between resilience and psychological distress in the general population is shown in Figure 3. For the general population subgroup, a moderately significant negative relationship was found between overall resilience and psychological distress (r = −0.45; 95% CI: −0.52 to −0.38; *p* < 0.001). The heterogeneity of the effect sizes was high (I^2^ = 99%).

#### 3.6.3. Patients

**Figure 4:**
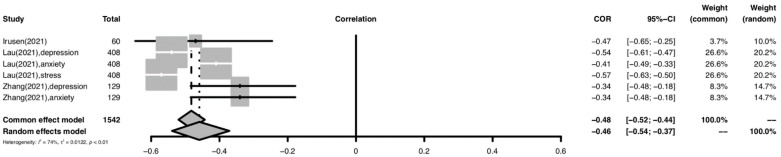
Forest plot of the relationship between resilience and psychological distress among patients Abbreviation: COR, correlation coefficient; CI, confidence interval; I^2^, indicator of statistical heterogeneity; *p*, *p*-value; $\tau^{2}$, indicator of statistical heterogeneity. Horizontal lines indicate the 95% CI of each study; diamonds indicate the pooled estimate with a 95% CI.

The forest plot for meta-analysis of the relationship between resilience and psychological distress among patients is shown in Figure 4. For the patient subgroup, a moderately significant negative relationship was found between overall resilience and psychological distress (r = −0.43; 95% CI: −0.52 to −0.33; *p* < 0.001). The heterogeneity of the effect sizes was high (I^2^ = 74%) but lower than those of the other subgroups.

#### 3.6.4. Publication Bias

**Figure 5:**
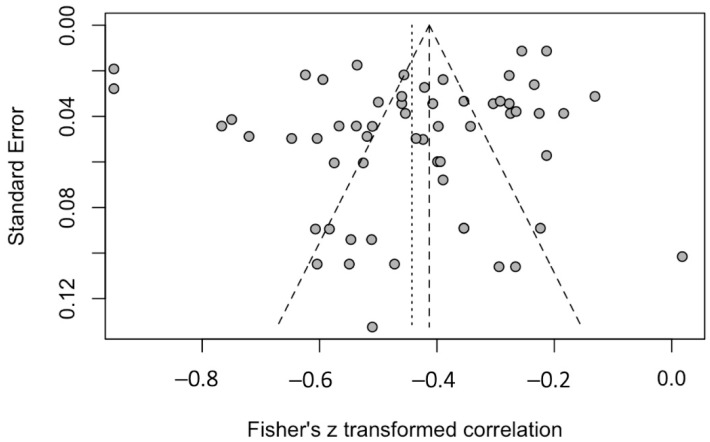
Funnel plot of effect sizes included in the meta-analysis.

The funnel plot for publication bias is provided in Figure 5. Based on publication bias analysis, visual evaluation of the funnel plot revealed that the distribution of the studies deviated from the funnel, which one would normally expect in the absence of publication bias. Therefore, this figure provides no visual indication of skewedness of the effect sizes observed. The left-sided test for funnel plot asymmetry using Egger’s regression test was not significant (*p* = 0.179), supporting the conclusion that no significant publication bias was present.

## 4. Discussion

### 4.1. Resilience and Psychological Distress

Based on the meta-analysis, we found a moderate negative relationship between resilience and psychological distress across populations during the COVID-19 pandemic (pooled r = −0.42; 95% CI: −0.45 to −0.38; *p* < 0.001). In other words, during the pandemic, the higher an individual’s resilience, the lower the psychological distress. The results indicate that resilience is essential in promoting a person’s positive mental health and reducing negative consequences. Our results align with a model of resilience hypothesizing that resilience supported mental health through risk reduction, protection, and promotion before the COVID-19 pandemic [82] and during the SARS pandemic [83]. More specifically, resilience reduces the depression, stress, anxiety, and PTSD associated with exposure to the COVID-19 pandemic. In addition, resilience appears to be a protective factor against adverse events and promotes a person’s ability to cope with COVID-19. Individuals with high resilience may have good tolerance of negative feelings, a strong capacity for self-reflection, and a high sense of responsibility, all characteristics that can promote better coping with psychological distress [84].

This relationship is similar to that found in previous systematic reviews and meta-analysis studies conducted before COVID-19. Färber and Rosendahl [29] reported a negative correlation of −0.43 (95% CI: −0.39 to −0.48; *p* < 0.001) between resilience and mental health problems in patients with a somatic illness or health condition. In addition, in the general population, Hu et al. [85] found that trait resilience was negatively correlated with negative indicators of mental health (mean effect size: −0.36, 95% CI: −0.37 to −0.35) and was positively correlated with positive indicators of mental health (mean effect size 0.53, 95% CI: 0.49 to 0.51). Furthermore, Joyce et al. [86] observed that a high level of resilience was associated with lower levels of anxiety, psychological distress, and depression.

Our results showed that the negative relationship between resilience and psychological distress was weaker among healthcare workers than in the general population and patients. Previous studies have shown that resilience among healthcare workers was lower than in the general population [87,88]. One explanation for these findings is that healthcare workers frequently experience stress and burnout and thus are more likely to have low resilience [89,90]. Based on the results, more attention should be focused on healthcare workers showing lower levels of resilience to help them better cope with the negative effects of mental health issues.

### 4.2. Healthcare Providers

Pooled analysis revealed that overall resilience was significantly negatively correlated with psychological distress among healthcare providers (pooled r = −0.39; 95% CI: −0.44 to −0.33; *p* < 0.001); however, the correlation was lower than pre-pandemic (r = −0.61) [91]. Studies reported that resilience was significantly negatively related to psychological distress among female nurses [92] and rescue workers [93]. A possible explanation for this association is that resilience can emerge as the ability to take full advantage of their positive personal characteristics despite stressful occupational circumstances. Moreover, healthcare providers’ experiences in providing care for COVID-19 patients and their family members, their access to expert colleagues and resources, their psychological knowledge, and their relatively high level of education [28] may help them to cope with psychological distress.

### 4.3. General Population

For the general population subgroup, a moderately significant negative relationship was found between overall resilience and psychological distress (pooled r = −0.45; 95% CI: −0.52 to −0.38; *p* < 0.001), which was somewhat weaker than the association found in a pre-pandemic study by Ghanei Gheshlagh et al. [30]. Those researchers found a moderate but stronger significant negative correlation between resilience and mental health issues in general populations (r = −0.54; 95% CI: 0.49 to 0.59; *p* = 0.0001). Other studies’ results also showed that the negative correlation between resilience and mental health in the elderly population and among business professionals pre-pandemic (r = −0.63 and −0.55, respectively) was greater than during the pandemic [94,95]. The weaker negative correlation between resilience and psychological distress observed in the general population during the pandemic may be attributable to their having less social support than previously. One attribute of resilience is social support [96], and given the preventive measures and isolation experienced during the pandemic, members of the general population may well have been denied contact with a supportive community of family members, friends, and coworkers. 

### 4.4. Patients

We found a moderately significant negative relationship between overall resilience and psychological distress in the patient subgroup (pooled r = −0.43; 95% CI: −0.52 to −0.33; *p* < 0.001). Similarly, a previous pre-pandemic study of somatically ill patients [29] reported that resilience was significantly negatively associated with psychological distress (r = −0.43; 95% CI: −0.48 to −0.39; *p* < 0.001). In addition, Cal et al.’s pre-pandemic study identified a significant negative relationship between resilience and mental health problems among patients with chronic illness [97]. In chronic illness patients, resilience may be a capacity that is developed over time in response to the stressors and hardships of contending with chronic disease. That is, compared to patients with acute illness, chronic illness patients tend to have higher resilience because in coping with their illness over the long term, they have time to adapt to their disease both physically and mentally [97]. 

### 4.5. Study Limitations

This study has limitations that should be noted. First, only research studies reporting correlational coefficients and employing the CD-RISC self-report measure were included, and only English-language publications were used; consequently, other relevant studies may have been inadvertently excluded. Second, only two of the included studies were cohort studies; the rest were cross-sectional and thus supplied only a snapshot of the existing situation with little or no longitudinal data. Third, the included studies measured psychological distress using various self-report measures and scales; given the heterogeneity of these measures, the pooled estimates should be interpreted with caution. Fourth, our search for relevant articles focused on publications from 1 December 2019 to 1 June 2021, but the first wave of the COVID-19 pandemic began and ended at various times, depending on the specific country and healthcare system involved. Fifth, we were unable to meaningfully compare the association between psychological distress and resilience before and after the COVID-19 pandemic, although based on previous systematic reviews, the association does not appear to have changed significantly. Future studies should examine the impact of COVID-19 on this association. Finally, this study focused on adult and older adult populations, and thus its findings may not be generalizable to child and adolescent populations. 

### 4.6. Research and Clinical Implications Format

This study’s findings shed light on the need to develop interventions for enhancing resilience among healthcare providers, the general population, and patients to decrease the long-term impacts of psychological distress. In clinical practice, these populations should receive psychosocial support during health emergencies such as COVID-19 and other infectious disease outbreaks. As an example, they could be provided with consultations with a psychologist to promote their resilience and reduce their psychological burden. Where healthcare providers are concerned, this approach might also reduce turnover rates and thus benefit the overall healthcare system. 

## 5. Conclusions

This systematic review and meta-analysis identifies a moderate negative relationship between resilience and psychological distress among healthcare workers, the general population, and patients in the COVID-19 context, although this association seems weaker than that found in the pre-pandemic period. In addition, this negative relationship was somewhat weaker among healthcare workers than was observed in the general population and patients. For all three populations, psychosocial support is needed to improve resilience and the ability to cope with psychological distress during the COVID-19 pandemic and in future disease outbreaks. On the whole, this study’s findings emphasize the need to develop specific interventions to enhance resilience in these populations.

## Figures and Tables

**Table 1:** Descriptive characteristics of and associations between resilience and psychological distress in the studies.

Study/Country	Sample	RS Version (Reliability)	Psychological Distress (Validity or Reliability)	N	*r* (Correlation Coefficient)	95% CI
Aruta [72] Philippines	Adult participants	CD-RISC-10 (α = 0.95)	Psychological distress (α = 0.90)	401	*r* = −0.40 ***	[−0.48; −0.31]
Awano et al. [73] Japan	Health care worker	CD-RISC-10 (-)	CESD (-) GAD-7 (-)	848	*r* = −0.27 *** r = −0.43 ***	[−0.33; −0.21] [−0.48; −0.37]
Doo et al. [74] Korea	Nurses	CD-RISC-10 (α = 0.94)	GAD-7 (α = 0.94) 9-items the depression screening tool Korean version (α = 0.94)	128	*r* = −0.525 ** r = −0.542 ***	[−0.64; −0.39] [−0.65; −0.41]
Ebrahimi et al. [71] Iran	Nurses	CD-RISC-25 (α = 0.90)	The Corona Anxiety (α = 0.89)	100	*r* = 0.018	[−0.18; 0.21]
Feng et al. [49] China	First-line rescuers	CD-RISC-10 (α = 0.94)	PSS (-) GAD-7 (α = 0.94) PHQ-2 (-) PC-PTSD-5 (-)	671	*r* = −0.425 *** r = −0.182 *** r = −0.268 *** r = −0.222 ***	[−0.49; −0.36] [−0.25; −0.11] [−0.34; −0.20] [−0.29; −0.15]
Goldbach et al. [75] USA	18 years old or older LGBTQ+	CD-RISC-10 (α = 0.86)	PROMIS-Anxiety (α = 0.94)	220	*r* = −0.371 **	[−0.48; −0.25]
Hou et al. [51] China	Health care workers	CD-RISC-25 (α = 0.96)	SCL-90 (α = 0.98)	1472	*r* = −0.23 ***	[−0.28; −0.18]
Hou et al. [50] China	Nurses	CD-RISC-25 (α = 0.98)	GAD-7 (α = 0.95)	701	*r* = −0.259 ***	[−0.33; −0.19]
Hu et al. [52] China	Frontline Nurses	CD-RISC-10 (α = 0.96)	Zung’s-SAS (α = 0.87) Zung’s-SDS (α = 0.88)	2110	*r* = −0.427 *** r = −0.554 ***	[−0.46; −0.39] [−0.58; −0.52]
Huang et al. [53] China	Medical staffs	CD-RISC-25 (α = 0.96)	The Chinese PSS (α = 0.87)	587	*r* = −0.635 ***	[−0.68; −0.58]
Irusen et al. [76] South Africa	Prostate Cancer male patients	CD-RISC-25 (-)	STAI-S (reliability = 0.86–0.95)	60	*r* = −0.47 ***	[−0.65; −0.25]
Jin et al. [54] China	Chinese undergrad student	CD-RISC-25 (α = 0.93)	DASS-21 (α = 0.88 for depression, 0.84 for anxiety)	847	DASS-Depression: *r* = −0.386 *** DASS-Anxiety: *r* = −0.295 ***	[−0.44; −0.33] [−0.36; −0.23]
Jacome et al. [77] Portugal	Physiotherapists	CD-RISC-25 (α = 0.89)	DASS-21 (α = 0.74 for depression, 0.85 for anxiety)	511	DASS-Depression: *r* = −0.470 *** DASS-Stress: *r* = −0.378 *** DASS-Anxiety: *r* = −0.330 ***	[−0.53; −0.40] [−0.45; −0.30] [−0.41; −0.25]
Kimhi et al. [69] Israel	Individuals	CD-RISC-10 (α = 0.92)	9-BSI (α = 0.86)	1346	*r* = −0.398 ***	[−0.44; −0.35]
Kocjan et al. [78] Slovenia	Slovene adults	CD-RISC-10 (α = 0.94)	PSS (α = 0.89)	2722	stress: *r* = −0.74 ***	[−0.76; −0.72]
Lau et al. [55] China	Person with chronic illness	CD-RISC-10 (α = 0.94)	DASS-21 (α = 0.73 for depression, 0.92 for anxiety)	408	depression: *r* = −0.54 *** anxiety: *r* = −0.41 *** stress: *r* = −0.57 ***	[−0.61; −0.47] [−0.49; −0.33] [−0.63; −0.50]
Levaot et al. [70] Israel	Adults	CD-RISC-10 (α = 0.88)	Peritraumatic distress (α = 0.88) Anxiety symptom (α = 0.93)	902	*r* = −0.340 *** *r* = −0.284 ***	[−0.40; −0.28] [−0.34; −0.22]
Li et al. [56] China	Healthcare Workers	CD-RISC-25 (α = 0.64–0.76)	COVID-19 stress (-)	309	*r* = −0.21 ***	[−0.31; −0.10]
Lin et al. [57] China	Medical workers	CD-RISC-25 (α = 0.93)	Hospital Anxiety and Depression Scale (HADS) (α = 0.70 for depression, 0.73 for anxiety)	116	anxiety: *r* = −0.498 depression: *r* = −0.471 *	[−0.62; −0.35] [−0.60; −0.32]
Lubian et al. [66] Spain	Pregnant woman	CD-RISC-10 (α = 0.85)	Edinburgh Postnatal Depression Scale (sensitivity 79% and specificity 95.5%) STAI (α = 0.82–0.95)	514	*r* = −0.491 ** STAI-State: *r* = −0.513 * STAI-Trait: *r* = −0.654 *	[−0.55; −0.42] [−0.57; −0.45] [−0.69; −0.59]
Morales-Vives et al. [67] Spain	Resident	CD-RISC-10 (IC: 0.88)	GHQ-12 (internal consistency = 0.76 for stress and 0.87 for overall score)	2055	GHQ-12:stress *r* = −0.27 **	[−0.31; −0.23]
Ou et al. [58] China	Nurses	CD-RISC-25 (α = 0.90)	Chinese version SCL-90 (α = 0.91)	92	Depression: *r* = −0.286 ** Anxiety: *r* = −0.260 *	[−0.46; −0.09] [−0.44; −0.06]
Pan et al. [59] China	Nurses and logistic staff	CD-RISC-25 (α = 0.91)	Chinese version PSS (α = 0.80) SAS (-)	423	*r* = −0.617 ** *r* = −0.477 **	[−0.67; −0.55] [−0.55; −0.40]
Pang et al. [60] China	Nurses	CD-RISC-25 (α = 0.76–0.91)	GAD-7 (Sensitivity and specificity ≥ 90%) PHQ-9 (Sensitivity and specificity > 90%)	282	*r* = −0.379 ** r = −0.375 **	[−0.47; −0.27] [−0.47; −0.27]
Schmitt et al. [79] Brazil	Brazilian adults	CD-RISC-25 (-)	PHQ-9 (-)	3274	*r* = −0.49 ***	[−0.52; −0.46]
Setiawati et al. [80] Indonesia	Healthcare workers	CD-RISC-25 (α = 0.87)	STAI (α = 0.91–0.94)	277	STAI-State: *r* = −0.519 *** STAI-Trait: *r* = −0.483 ***	[−0.60; −0.43] [−0.57; −0.39]
Tam et al. [61] China	HIV Healthcare Providers	CD-RISC-10 (α = 0.93)	PHQ-4 (α = 0.86) COVID-19 stressor (α = 0.78)	1029	*r* = −0.43 *** *r* = −0.13 ***	[−0.48; −0.38] [−0.19; −0.07]
Valero-Moreno et al. [68] Spain	Parents of adolescent	CD-RISC-10 (α = 0.92)	DASS (α = 0.87 for depression, α = 0.81 for anxiety, and α = 0.83 for stress)	94	depression: *r* = −0.44 ** anxiety: *r* = −0.54 *** stress: *r* = −0.50 ***	[−0.59; −0.26] [−0.67; −0.38] [−0.64; −0.33]
Wang et al. [1] China	Parent of children with autism spectrum disorder	CD-RISC-25 (α = 0.96)	SAS (α = 0.85) SDS (α = 0.87)	1764	Depression: *r* = −0.533 ** anxiety: *r* = −0.371 ***	[−0.57; −0.50] [−0.41; −0.33]
Xu et al. [62] China	Students	CD-RIS-27 (α = 0.86)	DASS (α overall = 0.882)	881	depression: *r* = −0.462 *	[−0.51; −0.41]
Ye et al. [63] China	Students	CD-RISC-10 (-)	CEDs (-)	1293	*r* = −0.74 ***	[−0.76; −0.71]
Ye et al. [64] China	Students	CD-RISC-10 (α = 0.96)	Acute Stress Disorder (ASD) (α = 0.94) COVID-19-Related Stressful Experience (α = 0.60)	7800	stress: *r* = −0.21 *** COVID-19 related to stress: *r* = −0.25 ***	[−0.23; −0.19] [−0.27; −0.23]
Zhang et al. [65] China	Patients with mild to moderate illness	CD-RISC-25 (α = 0.91)	PHQ-9 (Sensitivity 75% and specificity 90%) GAD-7 (Sensitivity and specificity ≥ 95%)	129	depression: *r* = −0.34 ** anxiety: *r* = −0.34	[−0.48; −0.18] [−0.48; −0.18]
Mean effect size				34,366	*r* = −0.42	[−0.45; −0.38]

Abbreviation: BSI: Brief Symptom Inventory, CD-RISC: Connor–Davidson Resilience Scale, CESD: Center of Epidemiologic Studies of Depression Scale, DASS-21: Depression, Anxiety, and Stress Scale-21, GAD-7: 7-item Generalized Anxiety Disorder Scale, GHQ-12: General Health Questionnaire-12, IC: Internal Consistency, PCL: The abbreviated PTSD Checklist, PC-PTSD-5: Primary Care Posttraumatic Stress Disorder Screen for DSM-5, PHQ: Patients Health Questionnaire, PROMIS-anxiety: The Patient Self-Report Outcome Measurement Information System-Anxiety, Symptom Checklist-90, PSS-10: Perceive Stress Scale-10, SAS: Self-rating Anxiety Scale, SCL: Symptom Checklist, SDS: Self-rating Depression Scale, STAI: State-Trait Anxiety Inventory. * *p* < 0.05; ** *p* < 0.01; *** *p* < 0.00.

## Data Availability

The data presented in this study are available on request from the corresponding author.

## Supplementary Materials

The following supporting information can be downloaded at https://www.mdpi.com/article/10.3390/ijerph192214854/s1, Table S1: Search strategies; Table S2: JBI critical appraisal checklist for analytical cross-sectional studies; Table S3: The quality appraisal for cohort studies; Table S4: Instruments to measure psychological distress.
