# Supplementary material for: The Association between Resilience and Psychological Distress during the COVID-19 Pandemic: A Systematic Review and Meta-Analysis
Source: Int J Environ Res Public Health. 2022 Nov 11;19(22):14854. doi: 10.3390/ijerph192214854 (PMC9690093; doi:10.3390/ijerph192214854)
Supplement: Supplementary file 1 [file ijerph-19-14854-s001.zip › ijerph-1963328-supplementary final.pdf]

International Journal of
Environmental Research and Public Health

MDPI

Supplementary Table S1. PICO keywords for search strategies.

PICO	Keywords
Population	"general population," "older adult*," "age*," "geriatric*," "gerontology," "pensioner*," "aging," "senior citizen*," "elder*," "senescent*," "older people," "elderly people," "adult*," "health care worker*," "nurse*," "physician*," "doctor*," "patient*," "medical team*"
Intervention or Exposure	"COVID-19," "coronavirus disease," "SARS-CoV-2"
Comparison	Not applicable
Outcome	"resilience," "psychological distress," "anxiety," "stress," "post-traumatic stress disorder, " "PTSD," "depression," "depressive symptom*"

Search date: June 2, 2021

Supplementary Table S2. JBI critical appraisal checklist for analytical cross-sectional studies. (n = 31)

Study	1. Were the criteria for inclusion in the sample clearly defined?	2. Were the study subjects and the setting described in detail?	3. Was the exposure measured in a valid and reliable way?	4. Were objective, standard criteria used for measurement of the condition?	5. Were confounding factors identified?	6. Were strategies to deal with confounding factors stated?	7. Were the outcomes measured in a valid and reliable way?	8. Was appropriate statistical analysis used?	Total
Aruta [1]	N	Y	Y	Y	Y	Y	Y	Y	7/8
Awano et al. [2]	Y	Y	Y	Y	Y	Y	Y	Y	8/8
Doo et al. [3]	Y	Y	Y	Y	Y	Y	Y	Y	8/8
Ebrahimi et al. [4]	N	Y	Y	Y	U	U	Y	Y	5/8
Feng et al. [5]	N	U	Y	Y	U	U	Y	Y	4/8
Goldbach et al. [6]	Y	Y	Y	Y	Y	Y	Y	Y	8/8
Hou et al. [7]	Y	Y	Y	Y	Y	Y	Y	Y	8/8
Hou et al. [8]	Y	Y	Y	Y	Y	Y	Y	Y	8/8
Hu et al. [9]	Y	Y	Y	Y	Y	Y	Y	Y	8/8
Huang et al. [10]	Y	Y	Y	Y	Y	Y	Y	Y	8/8
Jin et al. [11]	Y	Y	Y	Y	Y	Y	Y	Y	8/8
Jacome et al. [12]	Y	Y	Y	Y	Y	Y	Y	Y	8/8
Kimhi et al. [13]	Y	Y	Y	Y	Y	Y	Y	Y	8/8
Kocjan et al. [14]	N	Y	Y	Y	Y	Y	Y	Y	7/8
Lau et al. [15]	N	Y	Y	Y	Y	Y	Y	Y	7/8
Levaot et al. [16]	Y	Y	Y	Y	Y	Y	Y	Y	8/8
Li et al. [17]	Y	Y	U	Y	Y	Y	U	Y	6/8
Lin et al. [18]	Y	Y	Y	Y	Y	Y	Y	Y	8/8
Lubian et al. [19]	Y	Y	Y	Y	Y	Y	Y	Y	8/8
Morales-Vives et al. [20]	Y	Y	Y	Y	Y	Y	Y	Y	8/8
Ou et al. [21]	N	Y	Y	Y	Y	Y	Y	Y	7/8

Study	1. Were the criteria for inclusion in the sample clearly defined?	2. Were the study subjects and the setting described in detail?	3. Was the exposure measured in a valid and reliable way?	4. Were objective, standard criteria used for measurement of the condition?	5. Were confounding factors identified?	6. Were strategies to deal with confounding factors stated?	7. Were the outcomes measured in a valid and reliable way?	8. Was appropriate statistical analysis used?	Total
Pan et al. [22]	Y	Y	Y	Y	Y	Y	Y	Y	8/8
Pang et al.[23]	Y	Y	Y	Y	Y	Y	Y	Y	8/8
Schmitt et al. [24]	Y	Y	Y	Y	Y	Y	Y	Y	8/8
Setiawati et al. [25]	Y	Y	Y	Y	Y	Y	Y	Y	8/8
Tam et al. [26]	Y	Y	Y	Y	Y	Y	Y	Y	8/8
Wang et al. [27]	Y	Y	Y	Y	Y	Y	Y	Y	8/8
Xu et al. [28]	N	Y	Y	Y	Y	Y	Y	Y	7/8
Ye et al. [29]	U	Y	Y	Y	Y	Y	Y	Y	7/8
Ye et al. [30]	Y	Y	U	Y	Y	Y	Y	Y	7/8
Zhang et al. [31]	Y	Y	Y	Y	Y	Y	Y	Y	8/8

Supplementary Table S3. The quality appraisal for cohort studies. (n = 2).

Study	1.Were the two groups similar and recruited from the same population?	2.Were the exposures measured similarly to assign people to both exposed and unexposed groups?	3.Was the exposure measured in a valid and reliable way?	4. Were confounding factors identified?	5. Were strategies to deal with confounding factors stated?	6. Were the groups/ participants free of the outcome at the start of the study (or at the moment of exposure)?	7. Were the outcomes measured in a valid and reliable way?	8. Was the follow up time reported and sufficient to be long enough for outcomes to occur?	9. Was follow up complete, and if not, were the reasons to loss to follow up described and explored?	10. Were strategies to address incomplete follow up utilized?	11. Was appropriate statistical analysis used?	Total
Irusen et al. [32]	Yes	Yes	Yes	Yes	Yes	Yes	Yes	Yes	Yes	Yes	Yes	11
Valero-Moreno et al. [33]	Yes	Yes	Yes	No	No	Yes	Yes	Yes	No	No	Yes	7

Supplementary Table S4. Instruments used to measure psychological distress.

Variables	Measurements	Range of Reliability	K
Depression	-Patients Health Questionnaire-9 (PHQ-9) [23, 24, 31]	α > .90	3
	-Patient Health Questionnaire-4 (PHQ-4) [26]	α = 0.86	1
	-Patient Health Questionnaire (PHQ-2) [5]	Not reported	1
	- Center of Epidemiologic Studies of Depression Scale (CES-D) [2,29]	Not reported	2

Variables	Measurements	Range of Reliability	K
	-9-items the depression screening tool Korean version [3]	α = 0.94	1
	- Zung's Self-rating Depression Scale (SDS) [9, 27]	α = 0.87-0.88	2
	-Depression, Anxiety, and Stress Scale-21 (DASS-21) [11, 12, 15, 28, 33]	α = 0.88 (depression)	5
	-Hospital Anxiety and Depression Scale (HADS) [18]	α = 0.70 (depression)	1
	- Edinburgh Postnatal Depression Scale (EPDS) [19]	*= 79%; **= 95.5%	1
	- Chinese version of Symptom Checklist-90 (SCL-90) [21]	α = 0.91	1
Anxiety	-Zung Self-rating Anxiety Scale (SAS) [9, 22, 27]	Not reported	3
	-The Corona Anxiety Questionnaire [17]	α = 0.89	1
	-7-item Generalized Anxiety Disorder Scale (GAD-7) [2, 3, 5, ,8, 23, 31]	α = 0.94	6
	-Patient self-Report Outcome Measurement Information System(PROMIS)-Anxiety [23]	α = 0.94	1
	-State-Trait Anxiety Inventory State (STAI-S) [8, 26, 31]	α = 0.86-0.95	3
	- Depression, Anxiety, and Stress Scale-21 (DASS-21) [11, 12, 15, 28, 33]	α = 0.84 (anxiety)	5
	-Hospital Anxiety and Depression Scale (HADS) [18]	α = 0.73 (anxiety)	1
	- Chinese version of Symptom Checklist-90 (SCL-90) [7]	α = 0.91	1
	-Anxiety Symptoms Questionnaire [7]	α = 0.93	1
Stress	-Perceived Stress Scale-10 (PSS) [5, 10, 14]	Not reported	3
	-The Chinese perceived stress scale [22]	α = 0.87	1
	- Depression, Anxiety, and Stress Scale-21 (DASS-21) [11, 12, 15, 28, 33]	Not reported	5
	- COVID-19 Stress [17]	Not reported	1
	-General Health Questionnaire-12 (GHQ-12) [20]	IC = 0.87	1
	-COVID-19 Stressors [26]	α = 0.78	1
	-Acute Stress Disorder (ASD) [30]	α = 0.94	1
	-COVID-19 Related Stressful Experience [30]	α = 0.60	1
PTSD	-Primary Care Posttraumatic Stress Disorder Screen for DSM-5 (PC-PTSD-5) [5]	Not reported	1
	-Peritraumatic distress disorder [16]	α = 0.88	1
Psychological Distress	-Psychological distress questionnaire [1]	α = 0.90	1
	- Symptom Checklist-90 (SCL-90) [7, 21]	α = 0.98	2
	-Brief Symptom Inventory (BSI) [13]	Not reported	1

*Sensitivity, **Specificity, IC: Internal consistency; abbreviation: K: Number of association .

References:

1. Aruta, J.J.B.R. Socio-ecological determinants of distress in Filipino adults during COVID-19 crisis. *Current Psychology* **2021**, 1-11.
2. Awano, N.; Oyama, N.; Akiyama, K.; Inomata, M.; Kuse, N.; Tone, M.; Takada, K.; Muto, Y.; Fujimoto, K.; Akagi, Y. Anxiety, depression, and resilience of healthcare workers in Japan during the coronavirus disease 2019 outbreak. *Internal medicine* **2020**, *59*, 2693-2699.
3. Doo, E.Y.; Kim, M.; Lee, S.; Lee, S.Y.; Lee, K.Y. Influence of anxiety and resilience on depression among hospital nurses: A comparison of nurses working with confirmed and suspected patients in the COVID-19 and non-COVID-19 units. *Journal of Clinical Nursing* **2021**, *30*, 1990-2000.
4. Ebrahimi, H.K.; Amirmohamadi, M.; Esmaeilian, S.; Sohrabi, S.; Iranmanesh, S.; Sohrabi, Z.; Jafarnejad, S. The relationship between resilience and anxiety of Coronavirus disease (COVID-19) in the nurses of Ali Asghar Children's Hospital in Tehran, 2020. *Pak J Med Health Sci* **2020**, 1426-1434.
5. Feng, Z.; Xu, L.; Cheng, P.; Zhang, L.; Li, L.-J.; Li, W.-H. The psychological impact of COVID-19 on the families of first-line rescuers. *Indian J Psychiatry* **2020**, *62*, S438.
6. Goldbach, C.; Knutson, D.; Milton, D.C. LGBTQ+ people and COVID-19: The importance of resilience during a pandemic. *Psychol Sex Orientat Gend Divers* **2021**, *8*, 123.
7. Hou, T.; Zhang, T.; Cai, W.; Song, X.; Chen, A.; Deng, G.; Ni, C. Social support and mental health among health care workers during Coronavirus Disease 2019 outbreak: A moderated mediation model. *Plos one* **2020**, *15*, e0233831.
8. Hou, T.; Yin, Q.; Xu, Y.; Gao, J.; Bin, L.; Li, H.; Cai, W.; Liu, Y.; Dong, W.; Deng, G. The mediating role of perceived social support between resilience and anxiety 1 year after the covid-19 pandemic: Disparity between high-risk and low-risk nurses in china. *Frontiers in Psychiatry* **2021**, *12*, 666789.

9. Hu, D.; Kong, Y.; Li, W.; Han, Q.; Zhang, X.; Zhu, L.X.; Wan, S.W.; Liu, Z.; Shen, Q.; Yang, J. Frontline nurses' burnout, anxiety, depression, and fear statuses and their associated factors during the COVID-19 outbreak in Wuhan, China: A large-scale cross-sectional study. *EClinicalMedicine* **2020**, *24*, 100424.
10. Huang, L.; Wang, Y.; Liu, J.; Ye, P.; Cheng, B.; Xu, H.; Qu, H.; Ning, G. Factors associated with resilience among medical staff in radiology departments during the outbreak of 2019 novel coronavirus disease (COVID-19): A cross-sectional study. *Med Sci Monit* **2020**, *26*, e925669-925661.
11. Jin, L.; Hao, Z.; Huang, J.; Akram, H.R.; Saeed, M.F.; Ma, H. Depression and anxiety symptoms are associated with problematic smartphone use under the COVID-19 epidemic: The mediation models. *Child Youth Serv Rev* **2021**, *121*, 105875.
12. Jácome, C.; Seixas, A.; Serrão, C.; Teixeira, A.; Castro, L.; Duarte, I. Burnout in Portuguese physiotherapists during COVID-19 pandemic. *Physiother Res Int* **2021**, 26, e1915.
13. Kimhi, S.; Marciano, H.; Eshel, Y.; Adini, B. Resilience and demographic characteristics predicting distress during the COVID-19 crisis. *Soc Sci Med* **2020**, *265*, 113389.
14. Kocjan, G.Z.; Kavčič, T.; Avsec, A. Resilience matters: Explaining the association between personality and psychological functioning during the COVID-19 pandemic. *Int J Clin Health Psycho* **2021**, *21*, 100198.
15. Lau, B.H.P.; Cheung, M.K.T.; Chan, L.T.H.; Chan, C.L.W.; Leung, P.P.Y. Resilience in the storm: Impacts of changed daily lifestyles on mental health in persons with chronic illnesses under the COVID-19 pandemic. *Int J Environ Res Public Health* **2021**, *18*, 5875.
16. Levaot, Y.; Greene, T.; Palgi, Y. The associations between media use, peritraumatic distress, anxiety and resilience during the COVID-19 pandemic. *J Psychiatr Res* **2020**, 145, 334-338
17. Li, W.-Q.; Yuan, P.; Sun, J.; Xu, M.-L.; Wang, Q.-X.; Ge, D.-D.; Jiang, M.-M.; Xing, L.-Q.; Du, W.-J.; Li, Q. Resilience, coping style, and COVID-19 stress: effects on the quality of life in frontline health care workers. *Psych, Health & Med* **2021**, 27, 312-324.
18. Lin, J.; Ren, Y.-H.; Gan, H.-J.; Chen, Y.; Huang, Y.-F.; You, X.-M. Factors associated with resilience among non-local medical workers sent to Wuhan, China during the COVID-19 outbreak. *BMC psychiatry* **2020**, *20*, 1-7.
19. Lubian Lopez, D.M.; Butrón Hinojo, C.A.; Arjona Bernal, J.E.; Fasero Laiz, M.; Alcolea Santiago, J.; Guerra Vilches, V.; Casaus Fernández, M.; Bueno Moral, A.; Olvera Perdigones, A.; Rodriguez Rodriguez, B. Resilience and psychological distress in pregnant women during quarantine due to the COVID-19 outbreak in Spain: A multicentre cross-sectional online survey. *J Psychosom Obstet Gynecol* **2021**, *42*, 115-122.
20. Morales-Vives, F.; Dueñas, J.-M.; Vigil-Colet, A.; Camarero-Figuerola, M. Psychological variables related to adaptation to the COVID-19 lockdown in Spain. *Frontiers in Psychology* **2020**, *11*, 2438.
21. Ou, X.; Chen, Y.; Liang, Z.; Wen, S.; Li, S.; Chen, Y. Resilience of nurses in isolation wards during the COVID**-** 19 pandemic: A cross-sectional study. *Psych, Health & Med* **2021**, *26*, 98-106.
22. Pan, W.; Hu, J.; Yi, L. Mental state of central sterile supply department staff during COVID-19 epidemic and CART analysis. *BMC Health Serv Res* **2020**, *20*, 1-9.
23. Pang, Y.; Fang, H.; Li, L.; Chen, M.; Chen, Y.; Chen, M. Predictive factors of anxiety and depression among nurses fighting coronavirus disease 2019 in China. *Int J Ment Health Nurs* **2021**, *30*, 524-532.
24. Schmitt Jr, A.; Brenner, A.M.; de Carvalho Alves, L.P.; de Almeida Claudino, F.C.; de Almeida Fleck, M.P.; Rocha, N.S. Potential predictors of depressive symptoms during the initial stage of the COVID-19 outbreak among Brazilian adults. *J Affect Disord* **2021**, *282*, 1090-1095.
25. Setiawati, Y.; Wahyuhadi, J.; Joestandari, F.; Maramis, M.M.; Atika, A. Anxiety and resilience of healthcare workers during COVID-19 pandemic in Indonesia. *J Multidiscip Healthc* **2021**, *14*, 1-8.
26. Tam, C.C.; Sun, S.; Yang, X.; Li, X.; Zhou, Y.; Shen, Z. Psychological distress among hiv healthcare providers during the COVID-19 pandemic in China: Mediating roles of institutional support and resilience. *AIDS and Behavior* **2021**, *25*, 9-17.
27. Wang, L.; Li, D.; Pan, S.; Zhai, J.; Xia, W.; Sun, C.; Zou, M. The relationship between 2019-nCoV and psychological distress among parents of children with autism spectrum disorder. *Glob Health* **2021**, *17*, 1-14.
28. Xu, Y.; Shao, J.; Zeng, W.; Wu, X.; Huang, D.; Zeng, Y.; Wu, J. Depression and creativity during covid-19: Psychological resilience as a mediator and deliberate rumination as a moderator. *Front Psychol* **2021**, 1-*13*.
29. Ye, B.; Zhou, X.; Im, H.; Liu, M.; Wang, X.Q.; Yang, Q. Epidemic rumination and resilience on college students' depressive symptoms during the COVID-19 pandemic: The mediating role of fatigue. *Front Pub Health* **2020**, *8*, 1-10.
30. Ye, Z.; Yang, X.; Zeng, C.; Wang, Y.; Shen, Z.; Li, X.; Lin, D. Resilience, social support, and coping as mediators between COVID-19-related stressful experiences and acute stress disorder among college students in China. *Appl Psychol: Health Well-Being* **2020**, *12*, 1074-1094.
31. Zhang, G.-Y.; Liu, Q.; Lin, J.-Y.; Yan, L.; Shen, L.; Si, T.-M. Mental health outcomes among patients from Fangcang shelter hospitals exposed to coronavirus disease 2019: An observational cross-sectional study. *Chronic Dis Transl Med* **2021**, *7*, 57-64.
32. Irusen, H.; Burger, H.; Fernandez, P.W.; Esterhuizen, T.; Suliman, S.; Seedat, S. COVID-19 related anxiety in men with localized prostate cancer at tertiary hospitals in the cape town, South Africa. *Cancer Control* **2021**, *28*, 1-6.
33. Valero-Moreno, S.; Lacomba-Trejo, L.; Tamarit, A.; Pérez-Marín, M.; Montoya-Castilla, I. Psycho-emotional adjustment in parents of adolescents: A cross-sectional and longitudinal analysis of the impact of the COVID pandemic. *J Ped Nurs* **2021**, 59, 44-51
